# SSFP cine images early post-gadolinium improve detection of myocardial oedema in acute myocardial infarction compared to T2-weighted dark blood turbo spin echo images

**DOI:** 10.1186/1532-429X-14-S1-P27

**Published:** 2012-02-01

**Authors:** Winnie Chan, Charles Peebles, Stephen Harden, James Shambrook

**Affiliations:** 1 Radiology, Queen Elizabeth Hospital, Hong Kong, Hong Kong; 2 Cardiothoracic Radiology, Southampton University Hospitals NHS Trust, Southampton, UK

## Summary

We performed adenosine stress MRI in patients with a recent MI and assessed the utility of SSFP cine images early post-contrast to image myocardial oedema compared with standard T2-weighted sequences. Early post-contrast SSFP cine images were able to reliably demonstrate tissue oedema.

## Background

Demonstration of myocardial oedema following MI allows differentiation of acute and chronic MI, and assessment of the area at risk of infarction. T2-weighted dark blood TSE imaging for oedema is technically demanding and prone to artefacts.

Our protocol for patients with acute MI include standard oedema imaging sequences and more recently T2 prepared single shot SSFP sequences, followed by adenosine-stress perfusion imaging. The short axis SSFP cine stack is performed immediately after the perfusion study in an early post contrast phase (2-3 minutes). We observed that the cine stack in this protocol showed high signal in the infarct area correlating with the distribution of oedema on T2-weighted sequences. We hypothesized that SSFP-cine images early post contrast might be a more robust and time efficient alternative to T2-weighted imaging with TSE sequences.

## Methods

We analysed thirteen patients who underwent CMR following an acute coronary syndrome. We only included patients showing tissue oedema on short axis T2 TSE dark blood sequences. These images were compared with the corresponding selected slices in post-stress short axis SSFP-cine images and where available T2-prepared single shot SSFP images. Image analysis was performed using dedicated software, CMR42 (Circle CVI, Calgary, Canada). Left ventricular endo and epicardial contours were drawn manually on selected short axis slices. Myocardial oedema was calculated as >2SD from normal remote myocardium for each slice in both T2 TSE dark blood sequences and post-stress short axis SSFP-cine images.

## Results

Post-perfusion SSFP-cine images showed significantly larger myocardial oedema volume, and higher relative percentage of high T2 signal volume/myocardial mass than the corresponding T2 TSE images (table 1). T2 prepared SSFP sequences were also performed in seven of the patients. The post-stress SSFP-cine images showed no significant difference in oedema volume compared to the T2 prepared SSFP sequences.

**Table 1:** Results of T2-weighted dark blood TSE and Post-stress perfusion cine SSFP imaging

	T2-weighted dark blood TSE	Post-stress perfusion cine SSFP	p-value*
Oedema volume (ml)	21.7 ± 9.1	31.7 ± 15.4	0.0031
Oedema mass (g)	22.8 ± 9.5	33.2 ± 16.2	0.0031
Relative percentage of oedema volume/Myocardial mass (%)	26.1 ± 6.3	38.0 ± 9.4	0.0010
Oedema ratio relative to skeletal muscle	Median : 1.6 Range: 1.1 -3.3		

* p-value: two-tailed paired t-test.

## Conclusions

SSFP-cine images early post-contrast are capable of detecting myocardial oedema. The extent and volume of oedema correlates well with that demonstrated on T2-prepared single shot SSFP sequences but is overestimated compared with standard T2 TSE sequences. SSFP-cine images early post-contrast may be a robust and time efficient way to image myocardial oedema and warrants further investigation in a prospective study.

## Funding

Nil.

**Figure 1:**
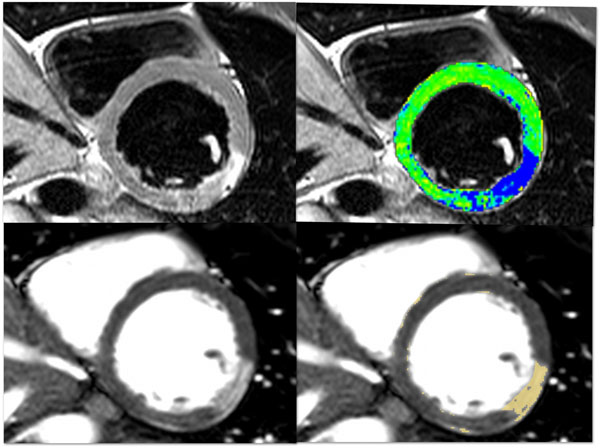
Top left: Short axis T2-weighted dark blood TSE image showed oedema in mid lateral wall of left ventricle. Top right: Short axis T2-weighted dark blood TSE image. Oedema ratio showed oedema in the mid lateral wall (blue) compared to the relative normal myocardium (green). Bottom left: Short axis post-gadolinium SSFP image showed corresponding oedema in mid lateral wall. Bottom right: Short axis post-gadolinium SSFP image highlighted the oedema area by semi-automated software using 2 standard deviation of mean normal myocardium.

